# Kisspeptin Exhibits Stimulatory Effects on Expression of the Genes for Kisspeptin Receptor, GnRH1 and GTH Subunits in a Gonadal Stage-Dependent Manner in the Grass Puffer, a Semilunar-Synchronized Spawner

**DOI:** 10.3389/fendo.2022.917258

**Published:** 2022-07-15

**Authors:** Md. Mahiuddin Zahangir, Md. Shahjahan, Hironori Ando

**Affiliations:** ^1^ Marine Biological Station, Sado Island Center for Ecological Sustainability, Niigata University, Niigata, Japan; ^2^ Department of Fisheries Management, Bangladesh Agricultural University, Mymensingh, Bangladesh

**Keywords:** GnIH, GnRH, GPR54, hypothalamus, gonadotropin, kisspeptin, puffer fish, reproduction

## Abstract

Kisspeptin has an important role in the regulation of reproduction by directly stimulating the secretion of gonadotropin-releasing hormone (GnRH) in mammals. In non-mammalian vertebrates, there are multiple kisspeptins (Kiss1 and Kiss2) and kisspeptin receptor types, and the two kisspeptins in teleosts have different effects depending on fish species and reproductive stages, serving reproductive and non-reproductive functions. In the grass puffer, *Takifugu alboplumbeus*, which has only a single pair of *kiss2* and *kissr2*, both genes display seasonal, diurnal, and circadian oscillations in expression in association with the periodic changes in reproductive functions. To elucidate the role of kisspeptin in this species, homologous kisspeptin peptide (gpKiss2) was administered at different reproductive stages (immature, mature and regressed) and the expression levels of the genes that constitute hypothalamo-pituitary-gonadal axis were examined in male grass puffer. gpKiss2 significantly elevated the expression levels of *kissr2* and *gnrh1* in the brain and *kissr2*, *fshb* and *lhb* in the pituitary of the immature and mature fish. No noticeable effect was observed for *kiss2*, *gnih*, *gnihr*, *gnrh2* and *gnrh3* in the brain and *gpa* in the pituitary. In the regressed fish, gpKiss2 was ineffective in stimulating the expression of the *gnrh1* and GTH subunit genes, while it stimulated and downregulated the *kissr2* expression in the brain and pituitary, respectively. The present results indicate that Kiss2 has a stimulatory role in the expression of GnRH1/GTH subunit genes by upregulating the *kissr2* expression in the brain and pituitary at both immature and mature stages, but this role is mostly ineffective at regressed stage in the grass puffer.

## Introduction

The reproduction in vertebrates is regulated by the complex interaction among multiple environmental factors and the reproductive neuroendocrine system, which is composed of kisspeptin, gonadotropin-inhibitory hormone (GnIH) and gonadotropin-releasing hormone (GnRH) in the hypothalamus and two pituitary gonadotropins (GTHs), namely follicle-stimulating hormone (FSH) and luteinizing hormone (LH) (1–5). Kisspeptin, a member of the RFamide peptide family, is encoded by the KISS1/Kiss1 gene in mammals. *KISS1* was originally identified as a metastasis suppressor gene (6) and its product was found to be the ligand for an orphan G-protein coupled receptor, GPR54, later named Kiss1r (7). Since mutations in *GPR54* were found to be responsible for idiopathic hypogonadotropic hypogonadism (8, 9), kisspeptin has received considerable attention as a potential key player in the neuroendocrine regulation of reproduction. It is well established in mammals that kisspeptin regulates reproductive events including puberty and ovulation through stimulating GnRH secretion (10).

Unlike most mammals that possess a single kisspeptin gene (*Kiss1*), most teleosts possess two paralogous genes for kisspeptin (*kiss1* and *kiss2*) and kisspeptin receptor (*kissr1*, *kissr2, kissr3* and *kissr4*) and this increases the complexity of the kisspeptin system in this group (11, 12). It has been shown that the role of the two kisspeptins in the regulation of reproduction varies among fish species. The administration of Kiss1 increased the plasma LH levels in goldfish (13) and stimulated gonadal development in chub mackerel (14). In striped bass, Kiss1 showed stimulatory effects on the expression of *fshb* and ovarian development, whereas Kiss2 exhibited no effect (5). In contrast, Kiss2 has been shown to have a stimulatory role in reproduction with higher potency when compared to Kiss1 in zebrafish, medaka, chub mackerel, European seabass, Nile tilapia and largemouth seabass (16–21). In addition, the actions of the two kisspeptins are different depending on the stage of gonadal development. In yellowtail kingfish, although only Kiss1 but not Kiss2 stimulated the expression of *fshb* and *lhb* during the breeding and non-breeding seasons, Kiss2 was more effective than Kiss1 in stimulating gonadal development during the non-breeding period (22). In hybrid bass, only Kiss2 was effective in stimulating LH secretion at puberty, whereas both Kiss1 and Kiss2 induced LH release at recrudescence stage (23). Moreover, Kiss2 upregulated *gnrh1* and *kiss2r* expressions in the brain at prepuberty, while it downregulated the expression of *gnrh1* and *kiss2r* at the recrudescence stage (23).

In contrast to this stimulatory role in most species depending on reproductive stage, it has recently been shown that the kisspeptin system is dispensable for reproduction in zebrafish (24–26) and medaka (27) using gene knockout models. Further studies suggest that this is because of physiological compensation that takes place in mutant fish to maintain reproductive processes (26, 28, 29). Moreover, co-expression of kisspeptin receptor in GnRH neurons has been controversial: co-expression has been demonstrated in Nile tilapia (30), African cichlid (31), striped bass (23), chub mackerel (32) and zebrafish (33), whereas the lack of co-expression has been shown in medaka (27, 34) and European sea bass (35). Taken together, the functional role and mode of action of kisspeptins in the control of reproduction in fish are controversial and varies depending on fish species and also reproductive stages.

The grass puffer, *Takifugu alboplumbeus*, shows unique reproductive physiology that is synchronized with the seasonal, lunar, and daily cycles (36, 37). During the spawning season from spring to early summer, spawning occurs on seashore only for several days around the new and full moon days every two weeks (36, 38). Mature fish usually aggregate for spawning at certain seashore locations 2–3.5 hours before high tide in the evening, and spawning occurs for 1.5–2 hours during the rising tidal phase. Therefore, the timing of spawning is tightly connected with seasonal, lunar, and tidal cycles as well as daily rhythm. Since the time and place of the spawning are known, spawning fish can be easily caught by dip net at the spawning bed. Thus, the grass puffer provides a unique animal model for studying the neuroendocrine mechanisms underlying the seasonal, lunar, and circadian controls of reproduction in wild animals.

Grass puffer has only a single pair of genes for kisspeptin (*kiss2*) and kisspeptin receptor (*kissr2*) and previous studies on their expression patterns with respect to seasonal, daily and circadian changes have indicated the possible importance of the kisspeptin system in the semilunar-synchronized spawning. The expression levels of both *kiss2* and *kissr2* show distinct changes during reproductive cycle with a significant increase from the early stage of gametogenesis to the pre-spawning and spawning stages (36, 39). This seasonal variations of *kiss2* and *kissr2* expressions are certainly important for the spawning in early summer and have recently been found to be regulated by water temperature: high water temperature conditions in summer (over 28°C) suppress the *kiss2* and *kissr2* expressions, leading to the termination of spawning period (40). Furthermore, *kiss2* and *kissr2* exhibit diurnal and circadian variations in expression during the spawning period (41). Therefore, the kisspeptin system is considered to be important in the stimulation, maintenance, and cyclicity of the reproductive function in the grass puffer.

In the present study, the effects of Kiss2 administration on the expressions of the genes for various hormones and receptors that are comprised in the hypothalamus-pituitary-gonadal (HPG) axis (*kiss2* and *kissr2*; *gnih* and *gnihr*; three GnRH genes, namely *gnrh1*, *gnrh2* and *gnrh3*; three GTH subunit genes, namely *gpa*, *fshb* and *lhb*) were examined to elucidate the functional significance of Kiss2 in the male grass puffer. For the possible different roles of Kiss2 during gonadal development, the fish were treated with Kiss2 at three reproductive stages, namely immature, mature and regressed stages.

## Materials and Methods

### Animals

Male fish with fully matured testes were collected from the spawning ground in Kawana, Shizuoka, Japan in June. Male fish with regressed testes were collected from the spawning ground in Minamiise, Mie, Japan at the end of July. The mature and regressed fish were transferred to the Marine Biological Station, Niigata University, Niigata, Japan, and reared in indoor tanks (500 L) with the flow of seawater under natural photoperiod (LD 14:10) for two weeks. The water temperature during the acclimatization period was similar to that of sampling ground, which was 20°C for the matured fish and 25°C for the regressed fish. The fish were fed daily with commercial pellets equivalent to 1% body weight (BW) until the experiment was conducted. Since immature grass puffer is unavailable from wild source, juvenile fish were artificially reared at the Fisheries Laboratory, University of Tokyo, Shizuoka, Japan, and they were transferred to the Marine Biological Station, Niigata University and reared in indoor conditions for one year. They were reared in indoor tanks (500 L) under natural photoperiod. Water temperature ranged from 16°C to 24°C depending on seasons. The experiment using the 1-year-old fish with immature testes was conducted in May.

### Kiss2 Administration

Grass puffer Kiss2 (gpKiss2, SKFNLNPFGLRFamide) (AB548304) was synthesized and dissolved in 0.9% NaCl and stored at -80°C until use. The fish were anesthetized in 0.008% tricaine methanesulfonate (MS222, Sigma-Aldrich, Tokyo, Japan) for 30 sec. and were immobilized with their ventral side upward. The immature and mature fish were intraperitoneally (ip) injected with gpKiss2 (0.1 and 1.0 μg/4 μl/g BW, n = 6−8) using a fine needle (25G, Terumo Corporation, Tokyo, Japan). Control groups of fish were injected with 0.9% NaCl. For the regressed fish, a preliminary experiment was conducted to examine the effect of gpKiss2 administration (0.01 and 0.1 μg/4 μl/g BW, n = 4) because there had been few reports on the effect of kisspeptin on animals at recrudescence stage. Since there was a trend toward increased *kiss2* and *gnih* expressions in the forebrain sample (telencephalon and diencephalon) at 0.01 μg/g BW in this preliminary experiment, the regressed fish were ip injected with gpKiss2 at 0.01 μg/g BW (n = 6−7) and the control fish were injected with 0.9% NaCl. In all experiments, the fish were injected at Zeitgeber time 2:00 (7:00 a.m.) and left in indoor tanks (100 L, n = 7−9 per tank) for 12 hrs.

### Sample Collection

The fish were anesthetized in 0.03% MS222 and total length and BW were recorded. Brains and pituitaries were removed after decapitation and soaked in RNAlater (Ambion, Austin, TX) at 4°C overnight. Gonads were removed and weighed for the calculation of gonadosomatic index (GSI = gonad weight/BW × 100). In the next day, brains were trimmed to prepare the forebrain sample that contained the telencephalon and diencephalon. The forebrain and pituitary samples were then stored at -80°C until RNA extraction. All the experimental procedures were carried out following the approved guidance by the Institutional Animal Care and Use Committee of the Niigata University, Niigata, Japan. Total length, BW, and GSI of the fish are shown in **Table 1**.

**Table 1:** Total length, body weight and gonadosomatic index (GSI) of fish samples. Values are presented as mean ± SEM.

Gonadal condition	No. of fish	Total length (cm)	Body weight (g)	GSI (%)
Immature	26	7.9 ± 0.1	8.7 ± 0.3	0.6 ± 0.1
Mature	24	14.9 ± 0.3	63.7 ± 0.3	16.3 ± 1.6
Regressed	13	13.4 ± 0.5	43.5 ± 5.1	1.1 ± 0.1

### Quantitative Real-Time PCR Assay

Real-time PCR assays for *kiss2*, *kissr2, gnih*, *gnihr*, *gnrh1*, *gnrh2*, *gnrh3*, *gpa*, *fshb* and *lhb* were carried out as described previously (39, 42, 43). Briefly, total RNA was extracted from the forebrain and pituitary samples and treated with DNase I (Takara, Ohtsu, Japan). Total RNA (200 or 500 ng) was used for synthesis of first strand cDNA using MultiScribe Reverse Transcriptase (Applied Biosystem, USA) and an oligo d(T)_12-18_ primer (2.5 μM) as per manufacturer’s instructions. The profile for reverse transcription reaction was 25°C for 10 min, 48°C for 30 min and 95°C for 5 min. The absolute amount of mRNA was determined using sense reference RNA, which was synthesized *in vitro* by a MAXIscript kit (Ambion) according to the manufacturer’s instruction and were serially diluted to 1 x 10^3^ − 1 x 10^8^ copies/μl. The standard sense RNAs were reverse transcribed and used as standard cDNAs to establish a standard curve. Real-time PCR was carried out with a Thermal Cycler Dice Real Time System III (TP970, TaKaRa Bio, Japan). PCR reaction mixture (10 μl) contained 1 μl of standard sample cDNA, 0.4 μl of forward and reverse primers (**Table 2**) and 5 μl of TB Green Premix DimerEraser (TaKaRa, Ohtsu, Japan). Amplification was carried out at 95°C for 30 sec, followed by 40 cycles at 95°C for 5 sec, 60°C for 30 sec and 72°C for 30 sec. Specific amplification of each cDNA was verified by melting curve analysis and gel electrophoresis of the product.

**Table 2:** Primers used in the real-time PCR assays in this study.

Primers	Nucleotide sequences
GnRH1-qPCR-F1	5́-CGGGAGTCTGATGTCACAGCTC-3́
GnRH1-qPCR-R1	5́-AACACTGACGACGACCGTGTCC-3́
GnRH2-qPCR-F1	5́-CAGGAGCTCACCTGTCCAAC-3́
GnRH2-qPCR-R1	5́-CTGCATTCTCCTGCTTCACAG-3́
GnRH3-qPCR-F1	5́-AAGCAAACAGGGTGATGGTG-3́
GnRH3-qPCR-R1	5́-CTGATGGTTGCCTCCAACTC-3́
GnIH-qPCR-F1	5́-TGATTCGTCTGTGCGAGGAC-3́
GnIH-qPCR-R1	5́-TCAGCAGCTGTGCATTGACC-3́
GnIH-R-qPCR-F1	5́-AAGATGCTCATCCTGGTGGC-3́
GnIH-R-qPCR-R1	5́-AGATCCACCTGGTCACTGTCC-3́
Kiss2-qPCR-F1	5́-GACCTTCAGGGACAACGAGGAC-3́
Kiss2-qPCR-R1	5́-ATGAAGCGCTTGCCAAAGC-3́
Kissr2-qPCR-F1	5́-TCCCGTTTCTGTTCAAGCACAAG-3́
Kissr2-qPCR-R1	5́-ATTGTTGTTGCGCTCCTCTGC-3́
GPα-qPCR-F1	5́-AAGGTGAGGAACCACACCGAG-3́
GPα-qPCR-R1	5́-AGCTCAAGGCCAGGATGAAC-3́
FSHβ-qPCR-F1	5́-ACACATTGAGGGCTGTCCAGTGG-3́
FSHβ-qPCR-R1	5́-TCCCCATTGAAGCGACTGCAG-3́
LHβ-qPCR-F1	5́-CACTTGGTGCAAACAAGCATC-3́
LHβ-qPCR-R1	5́-CAACTTAGAGCCACGGGGTAG-3́

### Statistical Analysis

To compare the effects of gpKiss2 administration on gene expression among various genes at three gonadal stages, the relative mRNA values with respect to control (0 μg/g BW) are expressed as mean ± standard error of the mean (SEM). Data were analyzed by ANOVA followed by Tukey’s HSD *post hoc* test to assess the statistically significant difference among different groups of immature and mature fish. Student t-test were performed to compare significant difference between the gpKiss2-injected and control groups in the regressed fish. Statistical significance was set at *p* < 0.05 unless described anywhere in the text. All statistical analyses were performed using SPSS Version 23.0 for windows (SPSS Inc., Chicago, IL).

## Results

### Effect of gpKiss2 on the Expression of *kiss2, kissr2, gnih, gnihr* and *three gnrhs* in the Brain of Immature and Mature Fish

**Figure 1:**
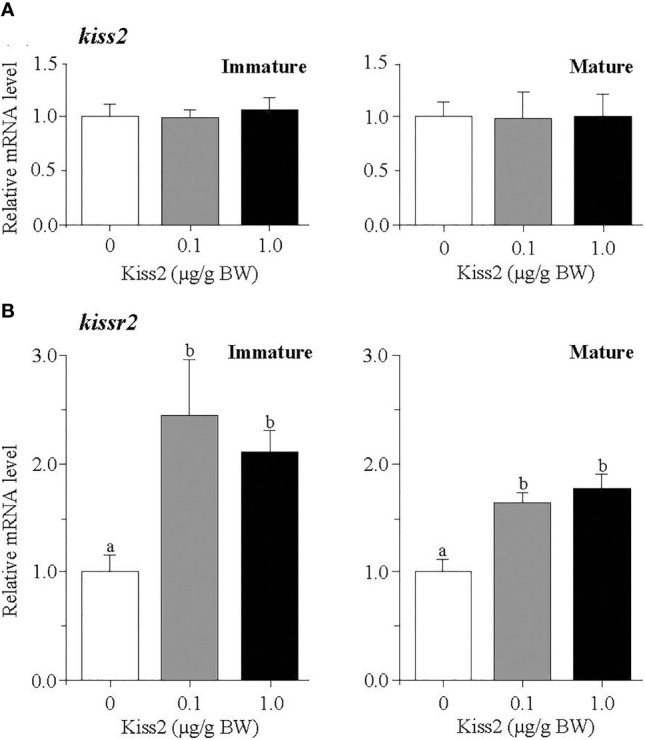
Changes in the relative mRNA levels of *kiss2* **(A)** and *kissr2* **(B)** in the brain of grass puffer at immature and mature stages after intraperitoneal injection of gpKiss2 for the period of 12hrs. Values are presented as mean ± SEM (n = 6−8). Values accompanied by different letters are statistically significantly different (*p* < 0.05).

**Figure 2:**
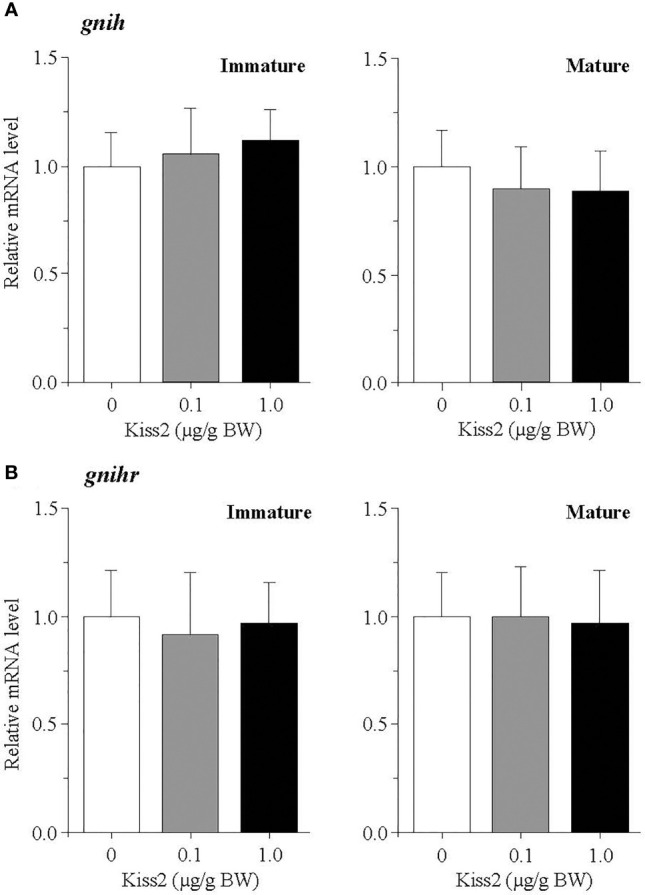
Changes in the relative mRNA levels of *gnih* **(A)** and *gnihr* **(B)** in the brain of grass puffer at immature and mature stages after intraperitoneal injection of gpKiss2 for the period of 12hrs. Values are presented as mean ± SEM (n = 6−8).

**Figure 3:**
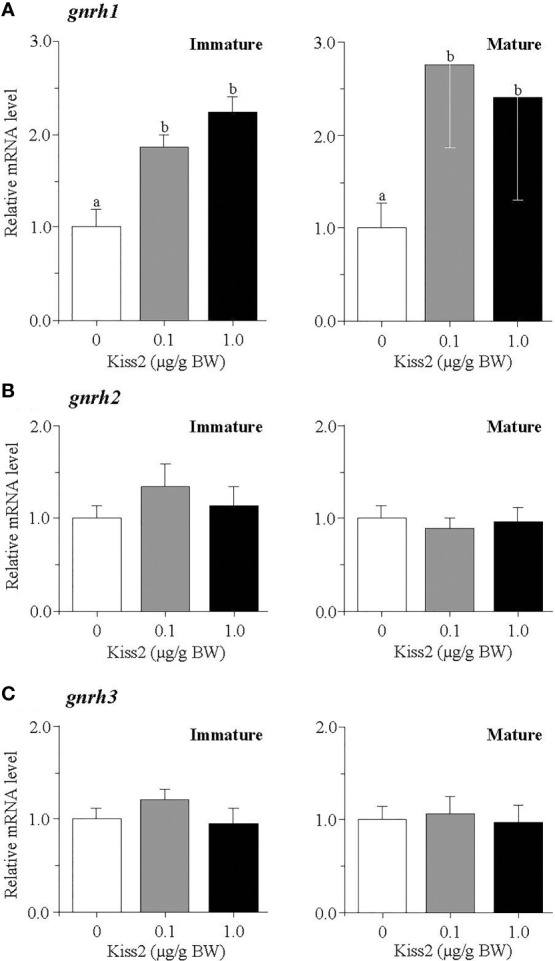
Changes in the relative mRNA levels of *gnrh1* **(A)**, *gnrh2* **(B)** and *gnrh3* **(C)** in the brain of grass puffer at immature and mature stages after intraperitoneal injection of gpKiss2 for the period of 12hrs. Values are presented as mean ± SEM (n = 6−8). Values accompanied by different letters are statistically significantly different (*p* < 0.05).

The administration of gpKiss2 did not alter the expression levels of *kiss2* in the immature and mature fish (**Figure 1A**). However, the expression of *kissr2* was significantly stimulated in the gpKiss2-injected fish at both immature and mature stages when compared to the control (**Figure 1B**). In contrast, gpKiss2 did not show any noticeable effect on the expression of *gnih* and *gnihr* in the brain of both immature and mature fish (**Figures 2A** , **B**). gpKiss2 significantly elevated the expression of *gnrh1* in the brain of immature and mature fish (**Figure 3A**). In the case of *gnrh2* and *gnrh3*, gpKiss2 did not show any effect at both immature and mature stages at any doses (**Figures 3B** , **C**).

### Effect of gpKiss2 on the Expression of *kissr2* and GTH Subunit Genes in the Pituitary of Immature and Mature Fish

**Figure 4:**
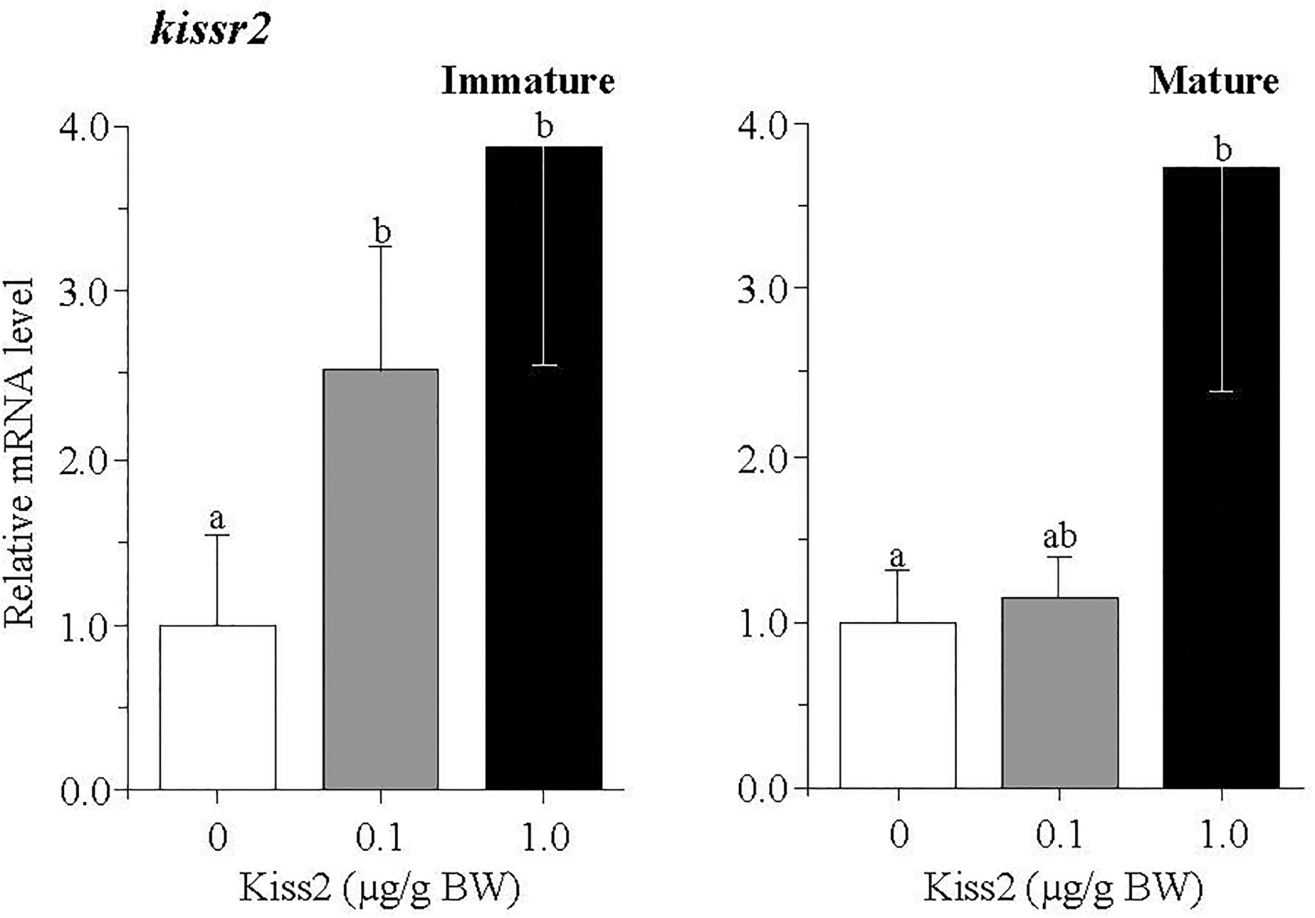
Changes in the relative mRNA levels of *kissr2* in the pituitary of grass puffer at immature and mature stages after intraperitoneal injection of gpKiss2 for the period of 12hrs. Values are presented as mean ± SEM (n = 6−8). Values accompanied by different letters are statistically significantly different (*p* < 0.05).

**Figure 5:**
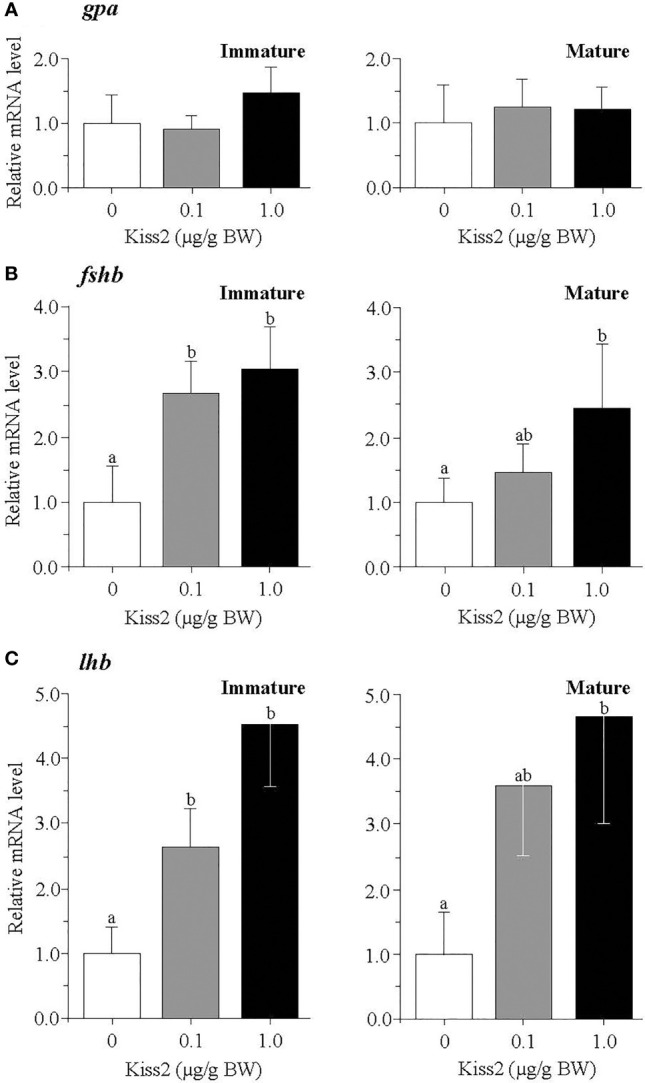
Changes in the relative mRNA levels of *gpa* **(A)**, *fshb* **(B)** and *lhb* ***(*C*)*** in the pituitary of grass puffer at immature and mature stages after intraperitoneal injection of gpKiss2 for the period of 12hrs. Values are presented as mean ± SEM (n = 6−8). Values accompanied by different letters are statistically significantly different (*p* < 0.05).

In the pituitary, the mRNA levels of *kissr2* were significantly increased by the gpKiss2 administration in both immature and mature fish and gpKiss2 showed higher potency to stimulate the *kissr2* expression in the immature fish compared to the mature fish (fold stimulation: immature 2.54 vs mature 1.14, *p* = 0.047 by t-test, **Figure 4**). Similarly, gpKiss2 significantly stimulated the expression of *fshb* and *lhb* in the immature and mature fish (**Figures 5B** , **C**), whereas no noticeable changes were observed for *gpa* (**Figure 5A**).

### Effect of gpKiss2 on the Expression of *kiss2, kissr2, gnih, gnihr* and *three gnrhs* in the Brain of Regressed Fish

**Figure 6:**
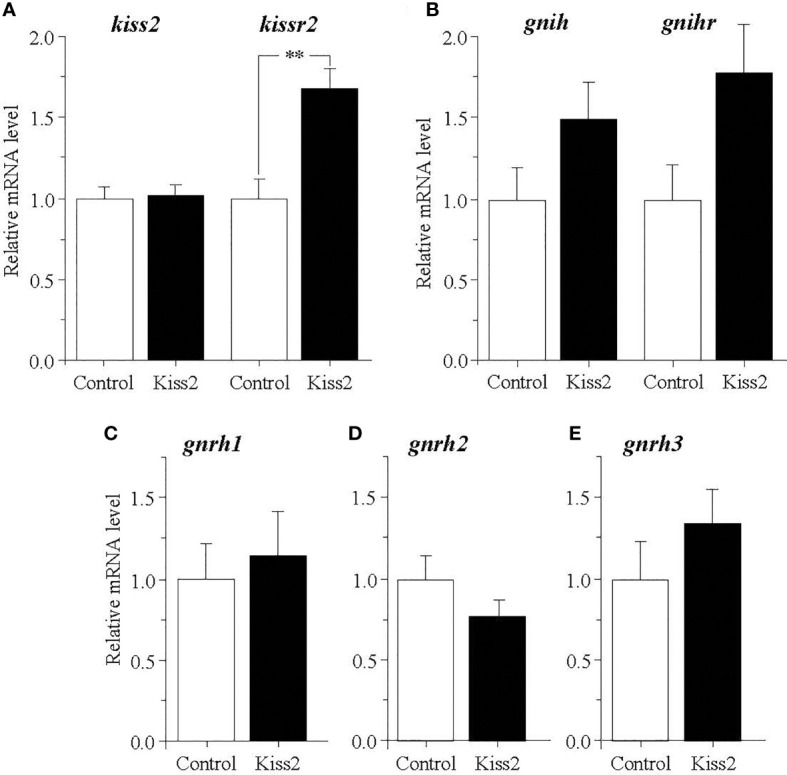
Changes in the relative mRNA levels of *kiss2* and *kissr2* **(A)**, *gnih* and *gnihr* **(B)**, *gnrh1* **(C)**, *gnrh2* **(D)** and *gnrh3* **(E)** in the brain of grass puffer at regressed stage after intraperitoneal injection of gpKiss2 for the period of 12hrs. Values are presented as mean ± SEM (n = 6−7). Asterisks denotes a significant difference between the control and gpKiss2 injected fish (**, *p* < 0.01).

In the brain of regressed fish, gpKiss2 did not show any change in the *kiss2* expression but significantly stimulated the expression of *kissr2* (**Figure 6A**). The expression levels of *gnih* and *gnihr* tended to be increased by the gpKiss2 administration, though these changes were not statistically significant (**Figure 6B**). There were no significant changes in the expression levels of three *gnrhs* after gpKiss2 administration in the regressed fish (**Figures 6C** –**E**).

### Effect of gpKiss2 on the Expression of *kissr2* and GTH Subunit Genes in the Pituitary of Regressed Fish

**Figure 7:**
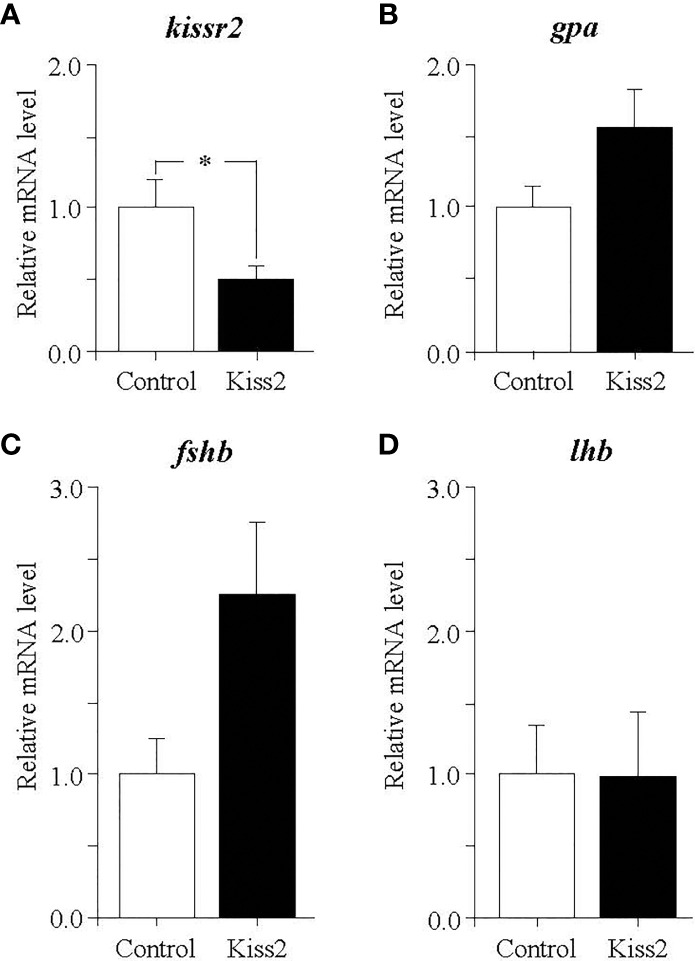
Changes in the relative mRNA levels of *kissr2* **(A)**, *gpa* **(B)**, *fshb* **(C)** and *lhb* ***(*D*)*** in the pituitary of grass puffer at regressed stage after intraperitoneal injection of gpKiss2 for the period of 12hrs. Values are presented as mean ± SEM (n = 6−7). Asterisks denotes a significant difference between the control and gpKiss2 injected fish (*, *p* < 0.05).

In the pituitary of regressed fish, gpKiss2 significantly decreased the *kissr2* expression (**Figure 7A**). There was a trend toward increased *gpa* and *fshb* expression by the gpKiss2 administration (**Figures 7B** , **C**) and no noticeable changes were observed in the *lhb* expression (**Figure 7D**).

## Discussion

The effect of gpKiss2 administration on the expression of the genes for the HPG axis was examined at three gonadal stages to elucidate the functional importance of the kisspeptin system in the male grass puffer. gpKiss2 significantly stimulated the expression of *kissr2* and *gnrh1* in the brain and *kissr2*, *fshb* and *lhb* in the pituitary of the immature and mature fish, showing a stimulatory role of gpKiss2 in reproduction by activating the *kissr2* expression in the brain and pituitary so as to stimulate the expression of GTHs. In the regressed fish, however, gpKiss2 was ineffective in stimulating the GnRH1 and GTH subunit gene expression, suggesting that the stimulatory role of gpKiss2 is dependent on the gonadal stage. Moreover, gpKiss2 did not alter the expression of *gnih*, *gnihr*, *gnrh2* and *gnrh3* as well as its own gene, *kiss2*.

In mammals, kisspeptin has a strong stimulatory effect on GTH secretion from the pituitary and this is mainly mediated through the stimulatory action on GnRH secretion (10, 29). Kiss1r is co-localized with GnRH neurons in the hypothalamus and the direct interaction between kisspeptin and GnRH is primarily important in the control of ovulatory cycle in mammals. In the present study, the effect of gpKiss2 was evaluated on the expression of three GnRH genes, *gnrh1*, *gnrh2* and *gnrh3* at three gonadal stages. In some teleost fish including the grass puffer, three GnRH neuronal groups are diversified in regard to localization and function (1, 5). GnRH1 neurons are mainly localized in the preoptic area (POA) and have a hypophysiotropic role through stimulating the GTH secretion from the pituitary. GnRH2 neurons are localized in the midbrain tegmentum and involved in appetite-related reproductive function (44–46). GnRH3 neurons are localized in the terminal nerve ganglion-POA region and have neuromodulatory action related to sexual behavior (47, 48). In the present study, gpKiss2 significantly stimulated the expression of *gnrh1* but not *gnrh2* nor *gnrh3*, with a concomitant increase in *kissr2* expression in the brain (**Figures 1B**, **3A–C**). Stimulatory action of kisspeptin on kisspeptin receptor gene expression has been reported in largemouth bass (21), yellowtail kingfish (22), and hybrid bass (23). In contrast, inhibitory action of Kiss2 on kisspeptin receptor gene expression was reported in the tongue sole (49). Although the co-localization of kisspeptin receptor with GnRH1 neurons remain to be determined in the grass puffer, *kissr2* mRNA was previously localized in the magnocellular preoptic nucleus pars magnocellularis (PMm) in the POA (41), which is one of the major hypothalamic nuclei that consist of hypophysiotropic neurons including GnRH1 neurons in many teleost species [for reviews (1) and (5)]. Co-expression of kisspeptin receptor in GnRH1 neurons has been reported in many fishes such as Nile tilapia (30), African cichlid (31), striped bass (23), chub mackerel (32) and zebrafish (33). Moreover, Kiss2 was shown to stimulate *gnrh1* expression *via* kisspeptin receptor using brain slices in striped bass (50). These and the present results suggest that gpKiss2 may directly activate GnRH1 neurons and stimulate the secretion of FSH and LH from the pituitary. Nevertheless, it is also possible that gpKiss2 could act on GnRH1 neurons indirectly *via kissr2*-expressing cells that are adjacent to GnRH1 neurons. In zebrafish, Kiss2 neurons project widely in the brain including the POA, where GnRH3 neurons are contacted by Kiss2 fibers (51). The immunolocalization of Kiss2 receptor (Kiss2-R) further showed in this species that, in addition to a subset of preoptic GnRH3 neurons were Kiss2-R-immunoreactive (ir), Kiss2-R-ir processes were observed in proximity to some GnRH3 neurons that were negative to Kiss2-R, suggesting that there is a possibility of direct and indirect actions of Kiss2 (33). Such close apposition of kisspeptin receptor and preoptic GnRH neurons has been reported in striped bass (23), European sea bass (35) and medaka (34). Taken as a whole, it is of considerable interest and importance to determine the neuroanatomical structure of Kiss2-R and GnRH1 neurons in grass puffer, which is currently under investigation.

In the pituitary, the augmented expressions of *fshb*, *lhb* as well as *kissr2* by gpKiss2 administration in the immature and mature fish (**Figures 4**, **5B, C**) suggest that gpKiss2 could have direct action on the regulation of pituitary. Stimulatory action of peripherally administered Kiss2 on GTH synthesis and release in the pituitary has been shown in goldfish (13), zebrafish (16), sea bass (17), hybrid bass (23), Nile tilapia (20), orange-spotted grouper (52) and seahorse (53). In addition, stimulatory effects of centrally administered Kiss2 on the *fshb* and *lhb* expressions were reported in chub mackerel (18). On the other hand, direct effect of Kiss2 has been examined using primary pituitary cultures, and the stimulatory effects were observed in sea bass (19) and zebrafish (54), whereas an inhibitory effect on *lhb* expression was reported in the primary culture of eel pituitary cells (55). The expression of kisspeptin receptor gene in the pituitary has been demonstrated in goldfish (13), European seabass (19), largemouth bass (21), yellowtail kingfish (22), zebrafish (54), eel (55), tongue sole (49), including grass puffer (39). In zebrafish, however, Kiss2-R (Kiss1Ra)-immunoreactivity was seen in corticotropes and melanotropes but not in gonadotropes (33), and the innervation of Kiss2 fibers to the *pars distalis* and also *pars nervosa* has been shown in striped bass (5), European sea bass (19, 35) and zebrafish (33, 54). These results suggest that Kiss2 may indirectly act on gonadotropes. Moreover, colocalization of Kiss2 cells with gonadotropes was observed in European sea bass (19). Taken together, these results indicate that Kiss2 has a local action on the pituitary in addition to the neuroendocrine action through preoptic GnRH neurons, and its action is most probably stimulatory in GTH secretion in many fishes including the grass puffer. Nevertheless, it should not be excluded in the present study that gonadal steroids are involved in the augmented expressions of *fshb* and *lhb* in response to the peripherally injected Kiss2 since kisspeptin and kisspeptin receptor are expressed in the gonads in many fishes including the grass puffer. The steroid feedback action on Kiss2 neurons and the pituitary gonadotropes needs be further examined.

The effect of gpKiss2 on the expression of *gnih* and *gnihr* was also examined in the present study. gpKiss2 did not show any significant changes in the expression of *gnih* and *gnihr* in the immature and mature fish (**Figure 2**). In the grass puffer, *gnih* and *gnihr* showed seasonal, daily and circadian variations like *kiss2* and *kissr2* (36, 37, 41, 42). GnIH administration experiments of grass puffer using a heterologous peptide (goldfish LPXRFamide) on the primary pituitary culture *in vitro* showed that goldfish LPXRFamide stimulated the expressions of *fshb* and *lhb* (42) as well as the genes for growth hormone and prolactin (56). These results suggest that GnIH is a multifunctional hypophysiotropic neurohormone, playing a stimulatory role in the control of reproduction in this species. Stimulatory and inhibitory effects of GnIH on reproduction have been reported in fish and the effects depend on the species and gonadal stages [for reviews (3) and (4)]. Although no changes in the *gnih* and *gnihr* expression in response to gpKiss2 administration were observed in the immature and mature fish, the *gnih* and *gnihr* mRNA levels tended to be higher in the gpKiss2-injected fish than the control fish at the regressed stage, suggesting that there may be a functional interaction between gpKiss2 and GnIH (**Figure 6B**). In the tongue sole, Kiss2 upregulated the expression of *gnih* and downregulated the expression of *gnihr* in the hypothalami in culture (49) and GnIH was shown to inhibit Kiss2-induced cAMP signaling in COS-7 cells transfected with both Kiss2-R and GnIHR expression vectors (57). It is tempting to speculate that Kiss2 and GnIH may have some functional interaction to stimulate GTH secretion in the grass puffer, in which GnIH positively regulates reproduction (37, 42, 56), and this needs to be further investigated.

In the regressed fish, there was no significant changes in the expression of *gnrh1*, *fshb* and *lhb* in the gpKiss2-injected fish (**Figures 6C**, **7C, D**), while *kissr2* expression was increased in the brain by the gpKiss2 injection but decreased in the pituitary (**Figures 6A**, **7A**). Although it is unclear why different response of *kissr2* expression was obtained in the brain and pituitary, the present results show that gpKiss2 is mostly ineffective in stimulating the expression of GnRH1/GTH subunit genes at the regressed stage. In yellowtail kingfish, Kiss1 administration significantly augmented the expression of pituitary *kiss2r* in the non-breeding season, whereas it was ineffective in increasing the *kiss2r* mRNA levels in the breeding season but was still effective in stimulating the *fshb* and *lhb* expressions (22). In hybrid bass, Kiss2 upregulated *gnrh1* and *kiss2r* expressions in the brain at prepuberty, while it downregulated the expression of *gnrh1* and *kiss2r* at the recrudescence stage (23). These results show that Kiss2 has a stimulatory role during gonadal maturation from prepubertal to mature stage in fish. In the grass puffer, the expression profiles of *kiss2* and *kissr2* during reproductive cycle show that both genes are activated at the early stage of gametogenesis and their expression levels are increased to the maximum levels at the breeding stages (36, 39). In chub mackerel, both *kiss1* and *kiss2* expressions were temporally increased at the onset of puberty and also during the breeding season (8). Increased expression of *kiss2* during the breeding season has also been reported in Senegalese sole (59) and European sea bass (60). Moreover, it has been reported that substantial increase in kisspeptin receptor gene expression occur before onset of puberty or during early puberty in zebrafish and medaka (16), Nile tilapia (20), cobia (61), grey mullet (62), flathead minnow (63), Atlantic halibut (64) and lined seahorse (53). Taken as a whole, these data show that the kisspeptin system is activated at the onset of puberty and also during the breeding stage in fish [see also review (2)]. The stimulatory effects of gpKiss2 on the expression of GnRH1/GTH subunit genes observed in the immature and mature fish in the present study are consistent with this notion.

In conclusion, gpKiss2 significantly stimulated the expression of *kissr2* and *gnrh1* in the brain and *kissr2*, *fshb* and *lhb* in the pituitary at immature and mature stages. The present results suggest that kisspeptin functions as a stimulatory neurohormone in the control of reproduction indirectly through preoptic GnRH1 neurons and directly by local action in the pituitary *via* the upregulation of *kissr2* expression. The stimulatory action of gpKiss2 on the expression of GnRH1/GTH genes depends on the reproductive stage, being mostly ineffective at the regressed stage in the grass puffer.

## Data Availability Statement

The raw data supporting the conclusions of this article will be made available by the authors, without undue reservation.

## Ethics Statement

The animal study was reviewed and approved by Institutional Animal Care and Use Committee of the Niigata University.

## Author Contributions

MMZ: design, experimentation, statistics, visualization, and writing. MS: design, experimentation, and statistics. HA: conception, design, writing, and supervision. All authors contributed to the article and approved the submitted version.

## Funding

This study was supported by MEXT/JSPS KAKENHI grants (26.04068, 16H04812 and 20H03288 to HA).

## Conflict of Interest

The authors declare that the research was conducted in the absence of any commercial or financial relationships that could be construed as a potential conflict of interest.

## Publisher’s Note

All claims expressed in this article are solely those of the authors and do not necessarily represent those of their affiliated organizations, or those of the publisher, the editors and the reviewers. Any product that may be evaluated in this article, or claim that may be made by its manufacturer, is not guaranteed or endorsed by the publisher.
